# Apolipoprotein C-I Levels Are Associated with the Urinary Protein/Urinary Creatinine Ratio in Pediatric Idiopathic Steroid-Sensitive Nephrotic Syndrome: A Case Control Study

**DOI:** 10.1155/2017/6392843

**Published:** 2017-01-30

**Authors:** Jun Odaka, Takahiro Kanai, Takane Ito, Takashi Saito, Jun Aoyagi, Hiroyuki Betsui, Takanori Yamagata

**Affiliations:** Department of Pediatrics, Jichi Medical University, 3311-1 Yakushiji, Shimotsuke, Tochigi 329-0498, Japan

## Abstract

Humoral factors may cause idiopathic steroid-sensitive nephrotic syndrome (ISSNS). In the present study, we analyzed serum proteins using mass spectrometry (MS) to identify proteins associated with the pathophysiology of pediatric ISSNS. We collected serial serum samples from 33 children during each ISSNS phase; Phase A1 is the acute phase prior to steroid treatment (STx), Phase A2 represents the remission period with STx, and Phase A3 represents the remission period after completion of STx. Children with normal urinalyses (Group B) and children with a nephrotic syndrome other than ISSNS (Group C) served as controls. No significant differences in urinary protein/urinary creatinine (UP/UCr) ratios were observed between the children with phase A1 ISSNS and Group C. We used surface-enhanced laser desorption/ionization time of flight MS for sample analysis. Four ion peaks with a mass-to-charge ratio (*m/z*) of 6,444, 6,626, 8,695, and 8,915 were significantly elevated during ISSNS Phase A1 compared to Phase A2, Phase A3, and Group C. The intensity of an* m/z* of 6,626 significantly correlated with the UP/UCr ratio and an* m/z* of 6,626 was identified as apolipoprotein C-I (Apo C-I). Apo C-I levels correlate with the UP/UCr ratio in pediatric ISSNS. Our findings provide new insights into the pathophysiology of ISSNS.

## 1. Introduction

Idiopathic steroid-sensitive nephrotic syndrome (ISSNS) is one of the most common chronic renal diseases in children and is caused by increased permeability of the glomerular filtration barrier [1]. A number of studies have suggested that the permeability factors underlying glomerular filtration barrier dysfunction are associated with the pathophysiology of ISSNS [2–5], but these factors have not yet been identified. Conventional serum proteomic analysis has the limitations of interference and charge suppression from high-abundance proteins, in addition to the hindrances caused by the extremely wide dynamic range (>10 orders of magnitude) of serum protein concentrations. Furthermore, the 22 most common protein species comprise approximately 99% of the total serum protein [6].

Surface-enhanced laser desorption/ionization time of flight mass spectrometry (SELDI-TOF MS) can overcome these problems. Using this technique, we have associated apolipoprotein A-II (Apo A-II) levels in serum with the status of ISSNS [7]; Apo A-II was found to induce type 2 helper T-cells (Th2) [7], which are predominantly found in the nephrotic phase in ISSNS [8]. In the present study, we tried to identify another protein associated with the status of ISSNS using SELDI-TOF MS.

## 2. Methods

This study was performed in accordance with the principles of the Declaration of Helsinki and was approved by the Ethics Committee of Jichi Medical University (A08-13, A14-055). Informed consent was obtained from all patients and/or their parents.

### 2.1. Patients and Controls

The study investigated 33 patients with ISSNS that were admitted to our department between March 2004 and November 2009. The study cohort consisted of three groups of children, those in Phase A1–A3 ISSNS and two age- and sex-matched control groups (Groups B and Group C) [7]. The characteristics of the groups are described in Table 1. The common inclusion criteria for the Phase A group were the presence of ISSNS, a selectivity index < 0.1 as determined by the immunoglobulin (Ig)G/transferrin clearance ratio, no infections for 1 week before the study began, and a 6-month absence of immunosuppressant therapy before the study began. Thirty-three patients were in the nephrotic phase of ISSNS before steroid therapy (STx) initiation (Phase A1), 33 patients were in the remission phase of ISSNS during STx (Phase A2), and 12 patients were in the remission phase of ISSNS after completing STx (Phase A3). The reason for fewer patients in Phase A3 is that 21 of 33 patients were STx-dependent.

Group B consisted of 15 healthy children with normal urinalyses, and Group C consisted of eight children with a nephrotic syndrome other than ISSNS (i.e., *n* = 1 with Alport syndrome, lupus nephritis, purpura nephritis, hemolytic uremic syndrome, IgA nephritis, or non-IgA mesangial proliferative glomerulonephritis; *n* = 2 with membranoproliferative glomerulonephritis type I).

No significant differences in the protein/urinary creatinine (UP/UCr) ratios (*P* = 0.26), the serum albumin (sAlb) level (*P* = 0.95), and the serum total cholesterol (T. chol.) level (*P* = 0.16) were found between Phase A1 and Group C. The sAlb level was significantly reduced in Phase A1 compared with Phase A3 (*P* < 0.05) or Group B (*P* < 0.05). The T. chol. level (*P* < 0.05) was significantly increased in Phase A1 compared with Phase A3 (*P* < 0.05). Student's *t*-test was applied to compare the differences between Phase A1 and each group. Anticholesterol agents had not yet been administered to any of the study participants.

### 2.2. Surface-Enhanced Laser Desorption/Ionization Time of Flight Mass Spectrometry

SELDI-TOF MS was performed according to the manufacturer's instructions with some previously described modifications [7].

### 2.3. Purification and Identification of ISSNS-Associated Peptides

Human serum samples (100 *μ*L) were diluted with 50 mM Tris buffer (pH 9.0) and loaded onto an anion-exchange column (Q Sepharose Fast Flow; GE Healthcare, Little Chalfont, UK). The samples were fractionated by applying buffer with declining pH values and the purification was confirmed using the Q10 ProteinChip® (Bio-Rad Laboratories, Inc., Hercules, CA). The desired fraction was diluted with 50 mM acetate buffer (pH 4.0) and loaded onto a cation-exchange column (CM Sepharose Fast Flow; GE Healthcare). The sample was fractionated by applying buffer with increasing sodium concentrations and the purification was confirmed using the NP20 ProteinChip (Bio-Rad Laboratories, Inc.). The desired fraction was separated by reverse-phase high-performance liquid chromatography (HPLC) (2 × 100 mm columns, TSK-GEL Super ODS; TOSOH, Tokyo, Japan) and the purification progress was confirmed using the NP20 ProteinChip (Bio-Rad Laboratories, Inc.). The desired fraction was purified using HPLC columns (2 × 150 mm) (TSK-GEL Amide-80; TOSOH, Tokyo, Japan), and the purification progress was monitored and it was confirmed using the NP20 ProteinChip (Bio-Rad Laboratories, Inc.). After a series of steps, we electrophoresed the desired fractions on Tris-tricine sodium dodecyl sulfate-polyacrylamide gels (polyacrylamide gel 16.2% T, 6% C) to evaluate the purity. Tandem mass spectrometry was used to analyze the purified fractions using a Nanoflow-LC ESI in positive mode (Q-TOF Ultima API®; Waters Corporation, Milford, MA, USA). Database searches were performed using Mascot® (Matrix Science Ltd., London, UK).

### 2.4. Serum Apolipoprotein C-I Measurements

Serum samples were collected from 11 patients in the Phase A1 subgroup and seven individuals in Group C to determine the serum Apo C-I levels. These two groups were matched for age and sex (Table 2). The two groups did not differ significantly with respect to their UP/UCr, serum albumin, and total cholesterol levels (*P* = 0.99, *P* = 0.21, and *P* = 0.33, resp.).

An enzyme-linked immunosorbent assay (ELISA) was performed to determine the Apo C-I levels in the serum samples from the two groups, using the Human Apolipoprotein C-I ELISA Kit (Assaypro LLC, MO, USA) according to the manufacturer's instructions. This assay employs a quantitative sandwich enzyme immunoassay technique. The standards and each sample were diluted to 100-fold concentration with diluent concentrate (EIA Diluent Concentrate; Assaypro LLC), and 50 *μ*L of each of the standards and samples was added to individual wells and the plate was incubated at room temperature for 2 h. After washing the plate with the wash buffer (Wash Buffer Concentrate; Assaypro LLC), 50 *μ*L of the biotinylated antibody (Biotinylated Apo C-I Antibody; Assaypro LLC) was added to each well, and the plate was incubated at room temperature for 2 h. After washing the plate with the wash buffer, 50 *μ*L of the streptavidin-peroxidase conjugate (Streptavidin-Peroxidase Conjugate, Assaypro LLC) was added to each well, and the plate was incubated at room temperature for 30 min. After washing the plate with the wash buffer, 50 *μ*L of a peroxidase enzyme substrate (Chromogen Substrate, Assaypro LLC) was added to each well, and the plate was incubated at room temperature for 30 min. After adding 50 *μ*L of stop solution (Stop Solution, Assaypro LLC) to each well, the absorbances were read by a microplate reader (Benchmark Plus Microplate Reader; Bio-Rad Laboratories, Inc.) at a wavelength of 450 nm.

### 2.5. Statistical Analyses

The data were analyzed using a two-tailed Kruskal-Wallis *H* test, followed by a two-tailed Mann–Whitney *U* test with Bonferroni correction to detect the peaks specific to the Phase A1 subgroup. We also analyzed the data to determine correlations between the intensities of each peak and the UP/UCr ratios using a two-tailed Spearman rank test.

Student's *t*-test was used to compare the differences in serum Apo C-I levels between the Phase A1 subgroup and Group C. *P* < 0.05 was considered significant.

## 3. Results

### 3.1. Detection of Peaks Associated with the Nephrotic Phase of ISSNS

**Figure 1:**
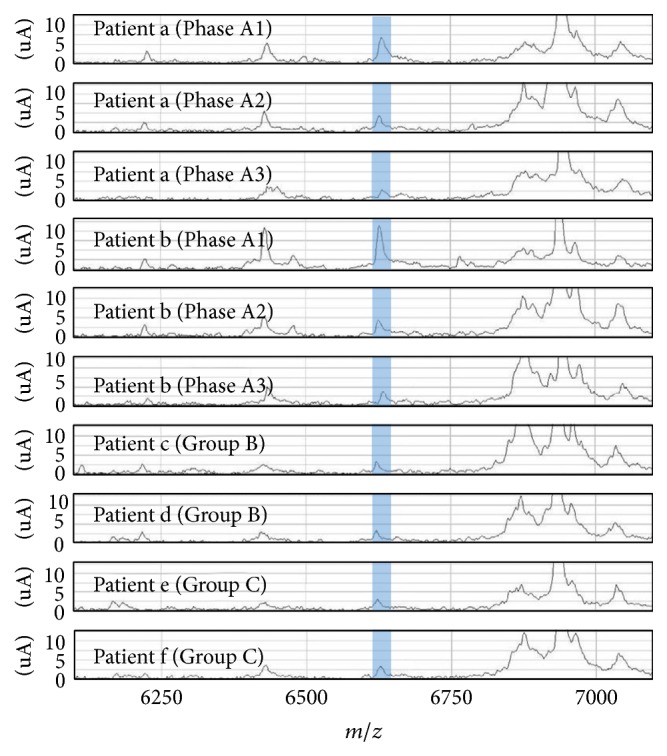
Representative spectra of six patients for the mass-to-charge ratio (*m/z*) of 6,626 (Q10, pH 5.0). The gray zone represents peaks at* m/z* 6,626. The upper six lanes show the serial change from Phase A1 to Phase A3 in patients a and b. The lower four lanes show the spectra of four patients from the control groups.

**Figure 2:**
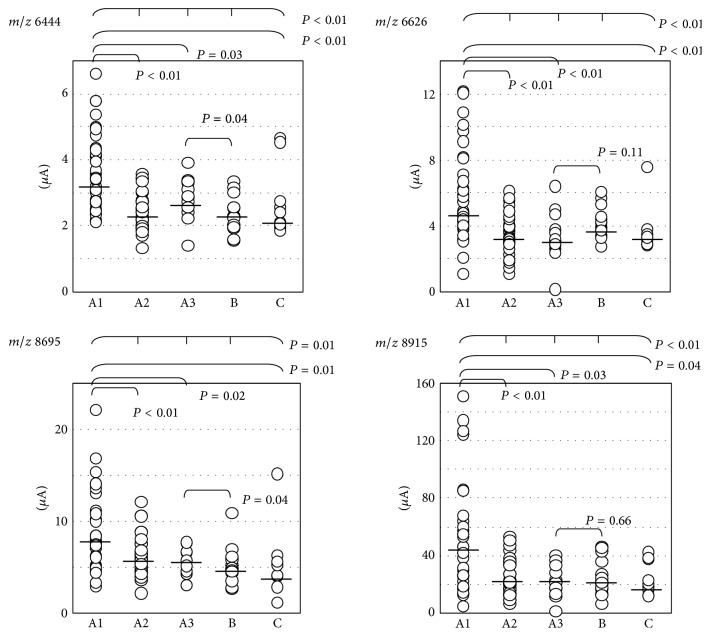
Intensity of each mass-to-charge ratio (*m/z*) level. Horizontal bars show each median value. The intensity of each* m/z* increased during Phase A1 [7].

Representative protein mass spectra for each ISSNS phase and Groups B and C are presented in Figure 1. To compensate for variations in the concentrations of the loaded samples, the peak intensities were normalized using a normal ion current and analyzed in Biomarker Wizard software (Bio-Rad Laboratories, Inc.). The data are presented as arbitrary units. The spectra of 101 samples exhibited 207 peaks with mass-to-charge ratios (*m/z*) between 2,000 and 10,000 using the following parameters for all spectra: first pass, 5.0; signal-to-noise ratio, 5.0 valley depth; minimum peak threshold, 5.0 valley depth; and minimum peak threshold 20.0%. Four peaks specific for Phase A1 were detected with an* m/z* of 6,444, 6,626, 8,695, and 8,915 (Figure 2). The intensities of these peaks were significantly greater for the Phase A1 subgroup than Phases A2 and A3 or Group C. No significant differences were observed between the patients in the Phase A3 subgroup and Group B with respect to the intensities of the peaks with an* m/z* of 6,626 or 8,915. The intensities of the peaks with an* m/z* of 6,444 and 8,695 were greater for the patients in the Phase A3 subgroup than those in Group B.

### 3.2. Correlations between Peak Intensities and UP/UCr Ratio

**Figure 3:**
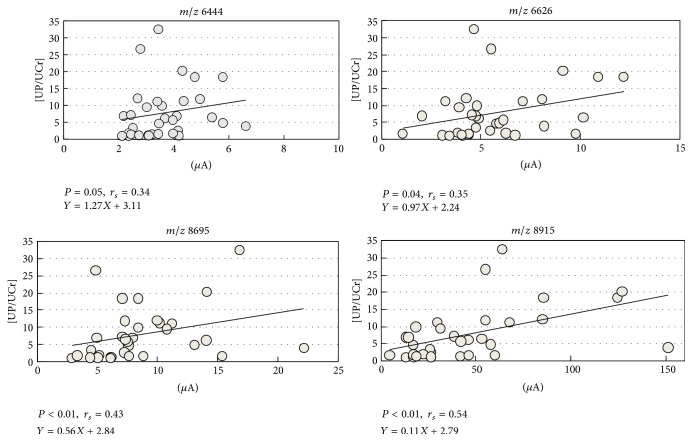
Correlations between the intensities of each mass-to-charge ratio (*m/z*) and each urinary protein/urinary creatinine (UP/UCr) ratio. Significant correlations were found between the intensities of the peaks at* m/z* ratios 6,626, 8,695, and 8,915 and their UP/UCr ratio [7].

Significant correlations were found between the intensities of the peaks with an* m/z* of 6,626, 8,695, or 8,915 and the UP/UCr ratios (Figure 3). The peak at* m/z* 6,626 was the only peptide ion with a *P* value < 0.01 between the Phase A1 subgroup and Phase A3 or Group C. Therefore, we tried to identify the original protein with an* m/z* of 6,626.

### 3.3. Mascot Identification of the Peak with* m/z* 6,626

**Figure 4:**
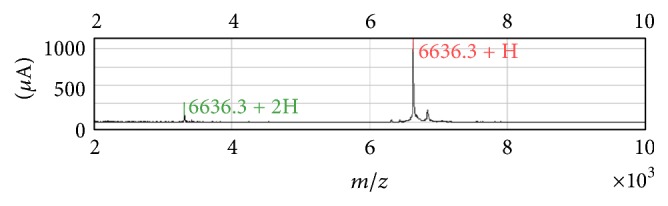
Peaks of mass-to-charge ratio (*m/z*) 6,626 after purification. The lower peak is the dication of* m/z* 6,626.

We identified the protein with an* m/z* of 6,626, the intensity of which correlated significantly with the UP/UCr ratio. We purified this protein from several mixed samples (Figure 4) and, by searching the Mascot database, were able to identify it as an Apo C-I fragment that has a score of 407. A score > 41 indicated identity or extensive homology (*P* < 0.05).

### 3.4. Serum Apo C-I Analysis

The mean serum Apo C-I levels were significantly higher in the Phase A1 subgroup than in Group C (87 ± 18 *µ*g/mL* versus* 64 ± 21 *µ*g/mL, *P* < 0.05). The mean serum Apo C-I levels in the Phase A1 subgroup were higher than the plasma reference value of 40–70 *µ*g/mL and were within the normal range in Group C.

## 4. Discussion

In the present study, we identified Apo C-I as a protein that is specifically elevated during Phase A1 of pediatric ISSNS, and its intensity correlated significantly with the UP/UCr ratio. In addition, the serum Apo C-I levels in Phase A1, which were determined by ELISA, were significantly higher than those found in Group C. Therefore, we conclude that this elevation is not secondary to the hyperlipidemia that is associated with nephrotic syndrome. To the best of our knowledge, these data are the first to suggest an association between Apo C-I levels and ISSNS status.

We suggest that Apo C-I may be associated with the pathophysiology of ISSNS.

One mechanism underlying this potential association is an increase in the serum Apo C-I levels reflecting the activation of macrophages during the nephrotic phase of pediatric ISSNS. Apo C-I is the smallest apolipoprotein (6.6 kDa) identified to date and comprises 57 amino acids [9, 10].* Apo C-I* mRNA is upregulated when monocytes differentiate into macrophages [11]. Shalaby et al. [12] reported that levels of interleukin-18, which is largely regarded as a product of macrophages [13], are significantly higher during the active stage of pediatric ISSNS compared to remission and controls. In previous studies, we found significantly increased levels of serum macrophage inflammatory protein-1*β* (MIP-1*β*) during the nephrotic phase of pediatric ISSNS before STx and during the remission phase during STx compared to the remission phase after completing STx [14]. These results suggest that macrophages are activated during the nephrotic phase of ISSNS, which may increase the production of MIP-1*β*. On the other hand, Chen et al. [15] reported a decrease in the expression of CD14, a monocyte marker, on the surface of peripheral monocytes from pediatric ISSNS patients during active relapses. However, patients who had already completed STx were included in their study, so the findings did not precisely reflect the phenomena associated with the pathophysiology of pediatric ISSNS. Schlecker et al. [16] demonstrated that intratumoral injections of MIP-1*β* increase the number of tumor-infiltrating regulatory T-cells (Tregs), suggesting that MIP-1*β* recruits high numbers of Tregs, which may play a role in the pathogenesis of pediatric ISSNS [17]. Accordingly, macrophage activation during the nephrotic phase of ISSNS may increase MIP-1*β* production and induce remission through the recruitment of Tregs. Furthermore, the increase in serum Apo C-I levels during the nephrotic phase of pediatric ISSNS may reflect macrophage activation rather than hypoalbuminemia.

Another potential mechanism is Apo C-I interacting with Th2 cell responses. Patients with pediatric ISSNS have a high incidence of atopic diseases [18], and atopy is characterized by Th2-dominant immune mechanisms [19], suggesting that Th2 plays an important role in the pathogenesis of ISSNS in children [8]. Nagelkerken et al. [10] demonstrated that mice transgenic for the expression of human Apo C-I in the liver and skin spontaneously develop symptoms associated with atopic dermatitis. One of the possible pathogenic mechanisms was enhanced Th2 activity facilitating a loss of skin integrity. Similarly, Apo C-I overexpression may cause functional or structural abnormalities in podocytes, and these abnormalities could induce the development of Th2-dominant immune mechanisms, which may be associated with the pediatric ISSNS phases. In our previous study, we identified* m/z* of 8,695 as an Apo A-II fragment [7]. Apo A-II inhibits interferon-*γ* production in human CD4 T-cells [20] and could lead to Th2-dominant immune mechanisms. Apo C-I may work in cooperation with Apo A-II.

A third mechanism is CD80 expression in podocytes being influenced by Apo C-I. Mice given lipopolysaccharide (LPS) expressed CD80 in their podocytes, which leads to podocyte foot process effacement and proteinuria [21]. These findings suggest that innate immunity to LPS may be involved in the pathogenesis of minimal-change nephrotic syndrome (MCNS). Berbé et al. [22] demonstrated that Apo C-I binds to LPS, which enhances the LPS-binding inflammatory response. Thus, Apo C-I might facilitate the expression of CD80 in podocytes and may be involved in the pathogenesis of MCNS in children.

## 5. Conclusions

We detected elevated Apo C-I levels during the nephrotic phase of pediatric ISSNS, and the Apo C-I levels were associated with the UP/UCr ratio. Since the sample size of Group C in this study is small, whether the elevation in Apo C-I was a secondary response to the hyperlipidemia associated with nephrotic syndrome or not is open to discussion. Nevertheless, these findings are an interesting starting point for further investigations into the pathophysiology of ISSNS.

## Figures and Tables

**Table 1:** Characteristics of the study groups [7].

Group	Criteria	*N*	M : F	Median age (range), years	Mean UP/UCr (g/gCr)	Mean serum albumin (g/dL)	Mean T. chol. (mg/dL)	Mean serum Cr (mg/dL)	Mean eGFR (ml/min/1.73 m^2^)	Mean disease duration, months	Mean systolic blood pressure (mmHg)
Phase A1	Nephrotic phase in ISSNS before STx initiation	33	21 : 12	7 (1–15)	8.3 ± 7.5	2.5 ± 1.1	319 ± 145	0.36 ± 0.14	136 ± 21	17 ± 35	110 ± 12

Phase A2	Remission phase in ISSNS during STx	33	21 : 12	7 (1–15)		2.8 ± 0.9	328 ± 113	0.38 ± 0.14	126 ± 19		110 ± 10

Phase A3	Remission phase in ISSNS after completing STx	12	8 : 4	6.5 (2–13)		4.0 ± 0.4	155 ± 20	0.34 ± 0.15	150 ± 25		103 ± 11

Group B	Normal urinalysis	15	7 : 8	5 (1–13)		4.6 ± 0.2	176 ± 3	0.32 ± 0.13	138 ± 22		97 ± 11

Group C	Nephrotic phase in nephrotic syndrome other than ISSNS before STx initiation	8	3 : 5	10 (4–14)	5.2 ± 3.2	2.6 ± 0.5	211 ± 59	0.73 ± 0.65	111 ± 46	17 ± 45	119 ± 22

M: male; F: female; UP/UCr: urinary protein/urinary creatinine; T. chol.: total cholesterol; Cr: creatinine; eGFR: estimated glomerular filtration rate; ISSNS: idiopathic steroid-sensitive nephrotic syndrome; STx: steroid treatment.

**Table 2:** Characteristics of groups analyzed for serum apolipoprotein C-I.

Group	Criteria	*N*	M : F	Median age (range), years	Average UP/UCr
Phase A1	Nephrotic phase in ISSNS before STx initiation	11	7 : 4	7 (1–13)	10.7
Group C	Nephrotic phase in nephrotic syndrome other than ISSNS before STx initiation	7	4 : 3	10 (4–15)	10.6

M: male; F: female; UP/UCr: urinary protein/urinary creatinine; ISSNS: idiopathic steroid-sensitive nephrotic syndrome; STx: steroid treatment.
